# Ionic Liquid-Based Electrolytes for Supercapacitor and Supercapattery

**DOI:** 10.3389/fchem.2019.00272

**Published:** 2019-04-18

**Authors:** Linpo Yu, George Z. Chen

**Affiliations:** ^1^Department of Chemical and Environmental Engineering, Faculty of Science and Engineering, Key Laboratory of More Electric Aircraft Technology of Zhejiang Province, University of Nottingham Ningbo China, Ningbo, China; ^2^Department of Chemical and Environmental Engineering, Faculty of Engineering, University of Nottingham, Nottingham, United Kingdom

**Keywords:** supercapacitor, supercapattery, micro-supercapacitor, ionic liquids, electrolytes, interfaces

## Abstract

There is a strong desire to replace or complement aqueous and organic electrolytes by ionic liquids (ILs) in electrochemical energy storage (EES) devices to achieve high operating voltages and hence high energy capacity. ILs are regarded as the inherent and competitive electrolytes since they were introduced to the electrochemical research community because they can overcome many disadvantages of the conventional aqueous and organic electrolytes, such as narrow potential windows, volatility, and flammability. This paper reviews critically the recent literatures of IL-based electrolytes used in supercapacitor, supercapattery, and micro-supercapacitor. Supercapattery is a generic term for various hybrid devices combining the merits of rechargeable battery and supercapacitor and often shows capacitive behavior. Fundamentals of supercapattery are briefly explained with typical examples. Micro-supercapacitor falls in the same scope of supercapacitor and supercapattery and shares the same fundamental concerns besides topology or structure. The future of IL-based electrolytes for the capacitive EES devices are also prospected.

## Introduction

Electrochemical energy storage (EES) technologies are currently playing the dominant and prospective roles in the globe effort to tackle the challenges to renewable energy supply (Dutta et al., 2014). One of the challenges is to efficiently store and supply energy harvested from the renewable sources at affordable cost compared with the traditional non-renewable options. All successful EES devices charge (storage) and discharge (release) electric charges reversibly, but their charging-discharging mechanisms are different in how and where the charges are stored. Based on these differences, there are three main types of EES technologies: (1) rechargeable batteries, including redox flow batteries, (2) supercapacitors, also known as electrochemical capacitors, and (3) various hybrids of battery and supercapacitor which are called supercapattery and supercabattery which have been discussed in the previous reviews (Chae et al., 2012; Akinwolemiwa et al., 2015; Yu and Chen, 2016b; Chen, 2017; Xia et al., 2017; Akinwolemiwa and Chen, 2018). Rechargeable batteries are recognized for their high energy capacity, whilst supercapacitors are perceived to have high power capability and long cycle life measured against the common ground (Chen, 2013; Simon and Gogotsi, 2013). Either the batteries or supercapacitors alone cannot satisfy the current commercial needs based on the consumption of fossil fuels. On this condition, several EES hybrid devices have been proposed and demonstrated in many studies that combine a battery electrode and a supercapacitor electrode into one device. Such hybrid device is a supercapattery if it has a capacitive performance exhibiting an enhanced energy capacity or it is a supercabattery if its performance is close to that of a battery. Supercabattery commonly possesses higher power capability and longer cycle life than battery. The performance of the hybrid devices mainly depends on the pairing of electrode materials. Table 1 summarizes the electrode compositions of supercapacitor, supercapattery, supercabattery and battery based on the charge storage mechanisms of electrode materials. The performance metrics of the typical cells, especially the ones using IL-based electrolytes are also concluded.

**Table 1:** Summary of pairing the electrode materials of different charge storage mechanisms^*^ into supercapacitor, supercapattery, supercabattery, and battery, and the performance metrics of the representative cells using different electrolytes (Yu and Chen, 2016b).

**Device**		**Supercapattery**						
	**Supercapacitor**			**Hybrid**				**Battery**
	**EDLC**	**Pseudocapacitor**		**Capacitive Hybrid**		**Others(Supercabattery)**		
Electrode Material	NFCS	NFCS	CFS	NFCS	CFS	NFCS	CFS	NCFS
	**+**	**+**	**+**	**+**	**+**	**+**	**+**	**+**
	NFCS	CFS	CFS	NCFS	NCFS	NCFS	NCFS	NCFS
Specific energy (Wh kg^−1^)	102 (IL), 6.7 (aq.)	3.6	26.6	230	261	–	208.6	250
Max specific power (kW kg^−1^)	111.6	24.7	13	59	25	–	3	1.5
Cycling life (cycles)	>10,000	>5,000	>5,000	>1,000	>10,000	–	>1,000	<1,200
Electrolyte type	IL, aq.	aq.	aq.	IL	IL	–	organic	organic
References	Lewandowski et al., 2010; Hou et al., 2015	Zhou et al., 2012	Huang et al., 2015	Zhang F. et al., 2013; Zhang L. et al., 2013; Yu and Chen, 2016a	Ortaboy et al., 2017	–	Zhou et al., 2016	^**^

* NFCS, Non-Faradaic Capacitive Storage = Electrical Double Layer Capacitance) Storage; CFS, Capacitive Faradaic Storage = Pseudocapacitive Storage; NCFS, Non-Capacitive Faradaic Storage = Battery-Type Storage;

** *data from web: https://en.wikipedia.org/wiki/Lithium-ion_battery#cite_note-7. The colors represent different charge storage mechanisms and relevant devices*.

Electrolytes, normally in liquid phase, are indispensable parts in all types of EES devices. They do not only help conduct electricity by means of transporting ions and keep an electronic insulation between positive and negative electrodes (positrode and negatrode), but also play a key role in exploiting the potentialities of EES devices. Generally, aqueous electrolytes are of high ionic conductivity and operational safety, but the maximum charging voltage (MCV) of an aqueous cell is limited by the splitting voltage of water. There is a strong desire to replace aqueous electrolytes by organic ones to achieve higher MCVs because the energy capacity can be dramatically promoted by the increased MCVs as described in Equation (1).

(1) $$\begin{array}{r} W_{\max}=\frac{1}{2}CU_{\max}^{2} \end{array}$$

where *C* is the capacitance of a capacitive EES cell, *W*_*max*_ represents the maximum energy capacity of the cell, and *U*_*max*_ is the symbol of MCV. Nowadays, organic electrolytes have been widely used in commercial Li-ion batteries and supercapacitors. However, traditional organic electrolytes have several inevitable disadvantages, like maintenance difficulty (tedious purification processes for the volatile and flammable solvents), high environmental impact, high cost, safety issues, and relatively low ionic conductivity, each of which can compromise the application of capacitive EES devices. There is strong desire to develop a new kind of electrolytes that can overcome these disadvantages.

Ionic liquids (ILs) are pure liquid salts in nature. They are specially featured by their practically zero or negligible volatility, highly ionized environment, broad liquid temperature ranges, and wide operating voltage windows. These features have brought about unique opportunities, where ILs have been used as the electrolytes for electrolysis (Sun et al., 2005; Yu et al., 2007, 2013), thermochromic materials (Wei et al., 2008, 2009; Yu and Chen, 2014), and the electrolytes in capacitive EES devices (Akinwolemiwa et al., 2015; Guan et al., 2016; Yu and Chen, 2016a,b; Xia et al., 2017; Shahzad et al., 2018).

There are several reviews related to those three kinds of electrolytes for different devises (Xia et al., 2017), such as battery (Chen et al., 2018), supercapacitor (Shahzad et al., 2018), etc. This article intends to review the recent progress of the IL-based electrolytes for capacitive EES devices, including supercapacitor, supercapattery, and micro-supercapacitor. The opportunity and challenge of these IL-based electrolytes are prospected based on the current knowledge.

## Supercapacitors

Electrical double layer capacitors (EDLCs) are part of supercapacitors and they store charges on the surface of electrode materials in principle. When a cell voltage is applied, the ions electrostatic adsorbed at the electrode/electrolyte interface contribute the charges stored by EDLCs. It is perceived that there should be no chemical reaction in EDLCs, and the charge storage process is widely considered to be physical in nature. Such mechanism has been proved by the fact that an EDLC using porous carbon electrodes can output a very high power of 90 kW kg^−1^, but its energy capacity is limited to 2~8 Wh kg^−1^ (Stevenson et al., 2015). Ions are always solvated in a bulk solution, but less so when they are adsorbed at the electrode | electrolyte interface. Solvation effect cannot be neglected when investigating the relationship between the pore size and the specific capacitance of porous carbons in aqueous or organic electrolytes. ILs have been used in the studies of EDLCs to avoid the effect of solvation because ILs are purely comprised of cations and anions.

### Neat IL Electrolytes for Supercapacitors

**Figure 1:**
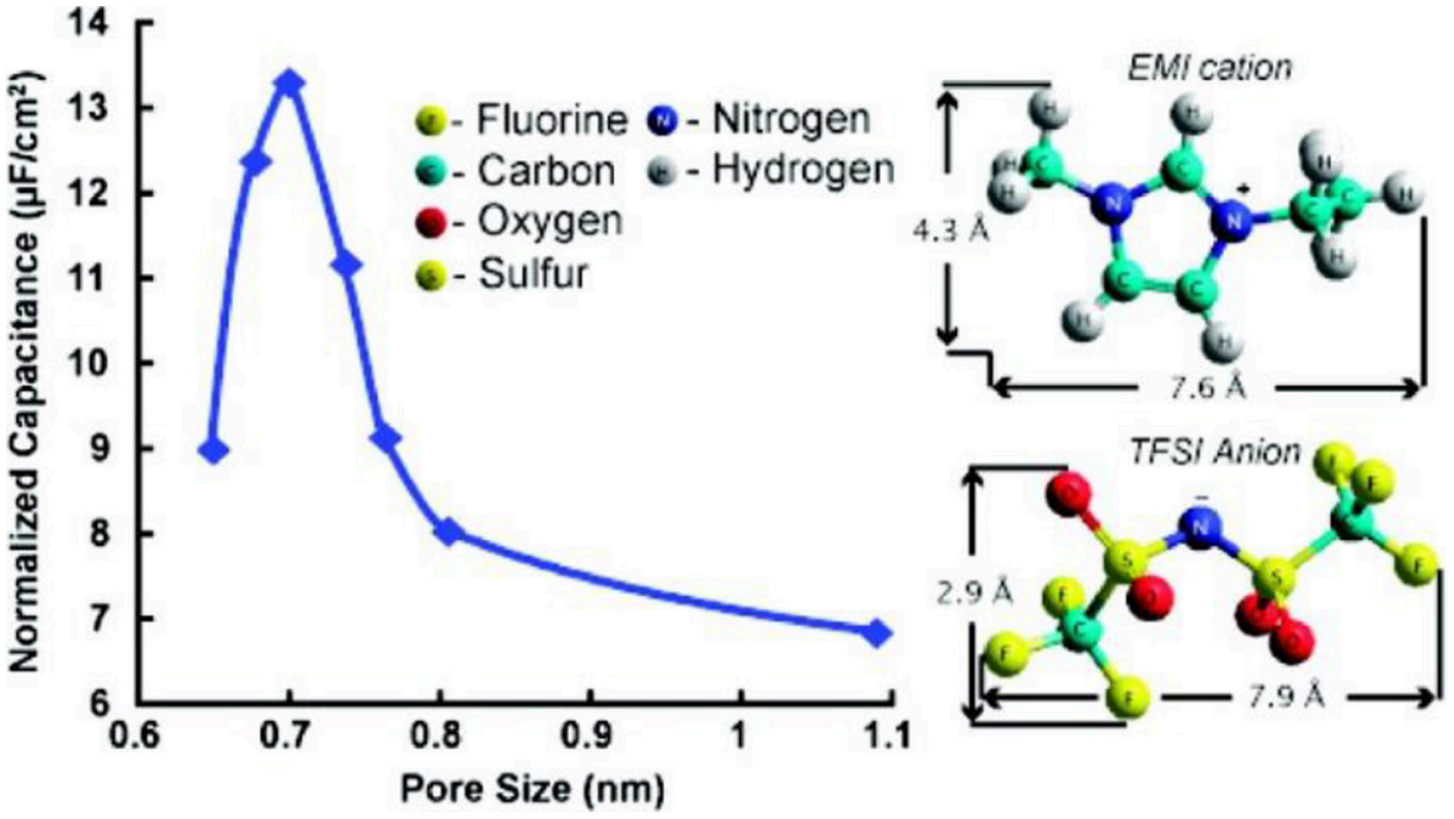
Specific area normalized capacitance changes *vs* the pore size of the carbide derived carbon (CDC) samples in ref (Largeot et al., 2008). (Reprinted with permission from American Chemical Society. Copyright 2008).

Amongst past attempts, an IL, 1-ethyl-3-methylimidazolium bis(trifluoromethylsulfonyl)imide (EMI-TFSI), was chosen to avoid solvation effect (Largeot et al., 2008). The longest dimensions of the EMI^+^ and TFSI^−^ ions are 0.76 and 0.79 nm, respectively, based on the calculation by using a HyperChem model. Because the average pore widths of carbide derived carbons (CDCs) can be easily modified from 0.65 to 1.1 nm by controlling the chlorination temperature from 400 to 1000°C, CDCs were used to fabricate the EDLC electrodes. Both EMI^+^ and TFSI^−^ ion sizes are within the range of the CDC pore sizes. Figure 1 clearly shows the pore size effect on the capacitance and the molecular structures of EMI^+^ and TFSI^−^ with the ion sizes corrected by using HyperChem. It was concluded that the maximum EDL capacitance could be achieved when the pore size of CDCs was very close to the ion size, suggesting that the ions entering sub nanometre pores would greatly promote the capacitance of EDLCs (Largeot et al., 2008). The mechanism of this enhancement was further studied in a molecular dynamic simulation of an IL, 1-butyl-3-methylimidazolium hexafluorophosphate (BMI-PF_6_), which was adsorbed inside the realistically modeled CDC electrodes (Merlet et al., 2012). The simulation showed the separated cations and anions inside the porous disordered carbons can yield a much higher capacitance than the ones with simple electrode geometries. A 3 V MCV was reached in the tests of these EDLCs and the specific capacitance reached 165 F g^−1^ (Largeot et al., 2008) and 125 F g^−1^ (Merlet et al., 2012) in EMI-TFSI and BMI-PF_6_, respectively.

It should be mentioned that the normalized capacitance in Figure 1 was calculated based on the specific capacitance and area of the CDCs. However, the area data were derived from the Brunauer-Emmet-Teller (BET) analysis (Largeot et al., 2008). IUPAC recommends the Non-Local Density Functional Theory (NLDFT) method for calculating the total surface area of microporous materials (Rouquerol et al., 1994) because this area is underestimated by using the BET method (Centeno and Stoeckli, 2010). The anomalous increase of the normalized capacitance below 1 nm pore size shown in Figure 1 was criticized afterwards by the other researchers who tested the different porous carbon materials in an organic electrolyte, tetraethylammonium tetrafluoroborate (TEA-BF_4_) in acetonitrile (AN) (Centeno et al., 2011). It was found that the surface-normalized capacitance of the different carbon samples is fairly constant from 0.7 to 15 nm pore size if the specific area was calculated by the NLDFT approach. Similar constant surface-normalized capacitance was found in a study of activated carbons (Act-Cs) in an organic electrolyte (Feng et al., 2010). Later, the NLDFT method has been always used when calculating the specific area of microporous carbons (Centeno and Stoeckli, 2011; Feng and Cummings, 2011; Hsieh et al., 2015; Galhena et al., 2016).

**Figure 2:**
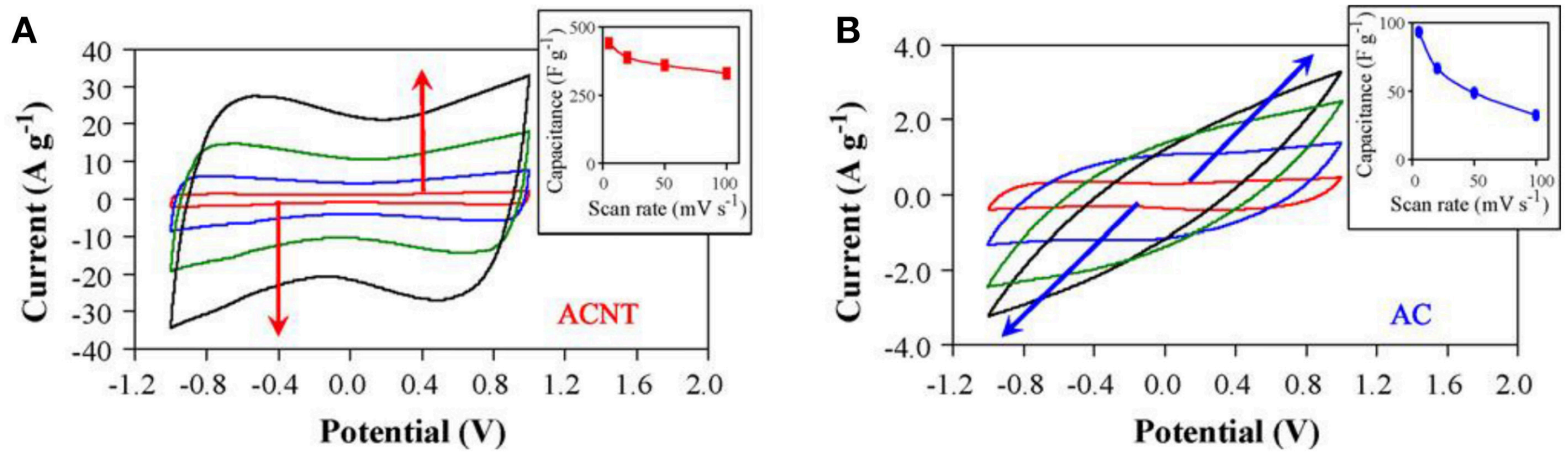
CVs obtained in EMI-TFSI for an ACNT electrode **(A)** and an Act-C electrode **(B)** at the scan rate increasing from 5, 20, 50, to 100mV s^−1^ as indicated by arrows. Insets show capacitance change of the electrodes upon increasing the scan rate (Lu et al., 2009). (Reprinted with permission from Elsevier. Copyright 2008).

Carbon nanotubes (CNTs) are another important type of carbon used in EDLC. Specifically, the vertically aligned CNTs (ACNTs) was used to further improve the rate capability and the MCV of EDLCs in IL electrolytes. The specific capacitance of ACNTs in an IL electrolyte, EMI-TFSI, was found to be 24 F g^−1^, which was lower than expected and approximate to the one of raw CNTs (Lu et al., 2009). However, the ACNTs after oxygen plasma etching presented a dramatically increased specific capacitance, up to 440 F g^−1^. These highly capacitive ACNTs were used as the electrode materials and EMI-TFSI was the electrolyte. The resultant supercapacitor has high cell voltage (up to 4 V), energy capacity (up to 148 Wh kg^−1^), and power capability (315 kW kg^−1^) (Lu et al., 2009). For comparison, Act-Cs were used as the reference materials in this work. Cyclic Voltammograms (CVs) of the ACNT and Act-C electrodes in the EMI-TFSI electrolyte were collected at different scan rates as shown in Figure 2. The MCV, energy capacity and power capability of this symmetrical supercapacitor are very promising even nowadays. It was noticed that the specific capacitance of ACNTs doubled the theoretical one based on the specific surface area, indicating there is another charge storage mechanism. Meanwhile, the ability of charge accumulation at the electrode | electrolyte interface strongly depends on the mesoporosity, pore size, and surface nature of CNTs and the accessibility of electrolyte. It was believed that the high capacitance in this IL should be related to the unique property of the plasma etched ACNTs. Pseudocapacitance can explain this phenomenon.

**Figure 3:**
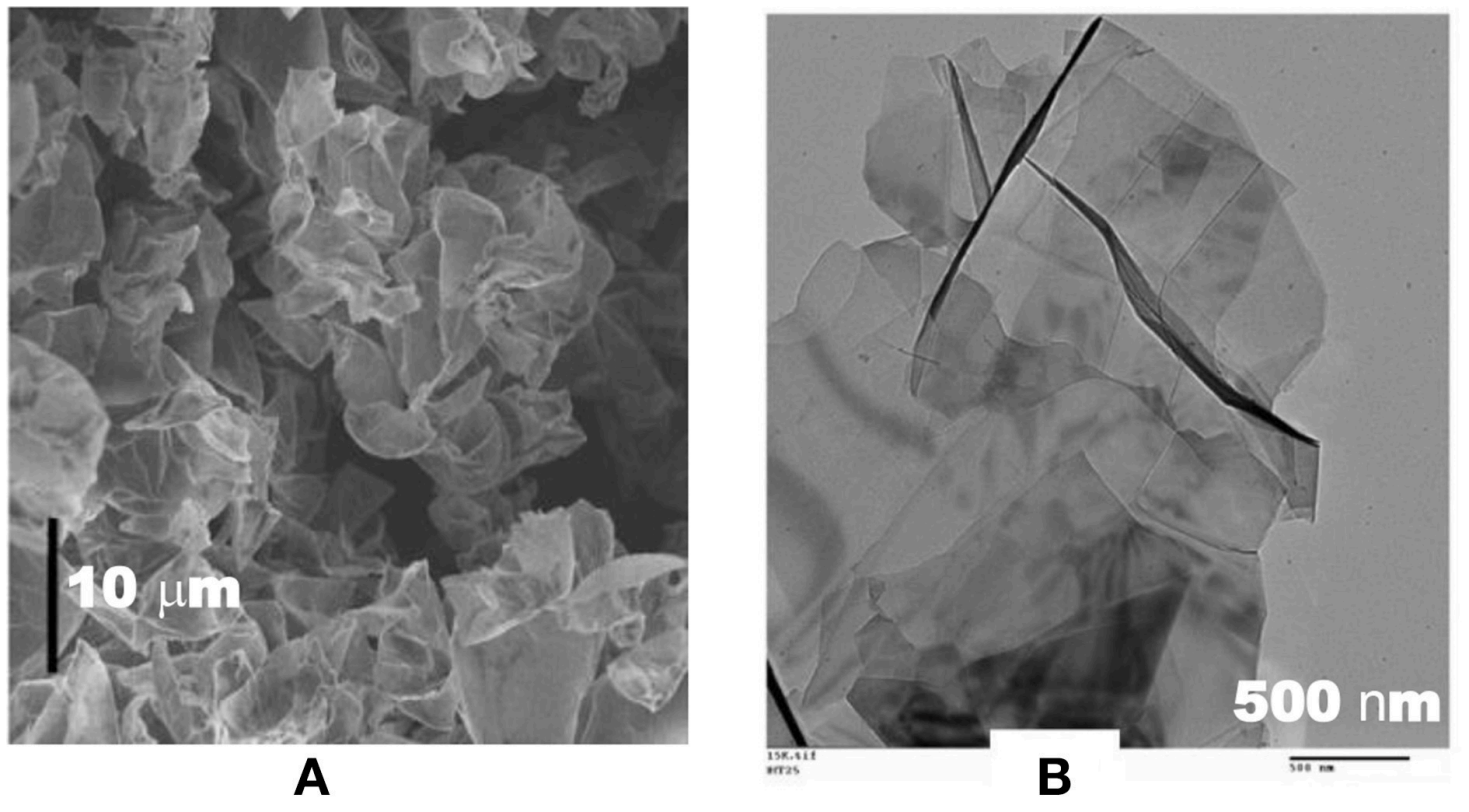
SEM image of curved graphene sheets (scale bar 10 μm) **(A)**, and TEM image **(B)** of flat graphene sheets prepared by a conventional chemical route (Liu et al., 2010). (Reprinted with permission from American Chemical Society. Copyright 2010).

Graphene possesses exceptionally a high specific surface area up to 2,675 m^2^ g^−1^. Like CNTs, graphene and its derivatives have attracted a great attention of the EDLC community. ILs have been used in the graphene based EDLCs to achieve high energy capacity. However, the reported specific energy of the graphene samples varies from one to another. Before explaining these phenomena, an important question should be raised that if the exceptionally large specific surface area of graphene has been fully utilized for the capacitance charge storage. For example, the specific capacitance of the curved graphene samples fell in the range of 10 to 250 F g^−1^ at 1 A g^−1^ in the EMI-BF_4_ electrolyte (Liu et al., 2010). It was explained that the morphology difference between the curved graphene sheets and the other conventional graphene sheets as shown in Figure 3 leads to the different specific capacitance, which ranges from 250 F g^−1^ to <10 F g^−1^ in the same IL electrolyte. Figure 3A shows a representative scanning electron microscopy (SEM) image of the curved graphene sheets, exhibiting a morphology that is favorable for preventing the graphene sheets from closely restacking with one another when they are packed or compressed into an electrode. In contrast, Figure 3B presents a typical transmission electron microscopy (TEM) image of the graphene sheets that were prepared by a conventional chemical route. The inter-graphene spacing is likely <1 nm as observed from Figure 3B. The TEM image also indicates that the flat graphene sheets tend to restack with one another, leading to a decrease of the effective surface area. A supercapacitor using the curved graphene sheets as the electrode materials and EMI-BF_4_ as the electrolyte exhibited a specific energy of 85.6 Wh kg^−1^ at 1 A g^−1^ at room temperature, and 136 Wh kg^−1^ at 80°C (Liu et al., 2010). It should be mentioned that the curved graphene sample shown in Figure 3 is actually a kind of reduced graphene oxide (r-GO) that contains many defects and oxygen containing groups (OCGs) on the surface. Perfect graphene in principle can only be prepared by a physical method and it is mostly favored by physicists. In most studies, the graphene-based materials consist of r-GOs that could be obtained from either the partial oxidation of graphene or, in the majority cases, the partial reduction of graphene oxide (GO). In the early studies, graphene and r-GO were used interchangeably by researchers, especially by chemists. Nowadays, r-GO is the preferred term if the sample is synthesized by the reduction of GO.

The study of the capacitance behavior of r-GO in ILs is still on-going. For example, GO could be partially reduced by a weak reductant, like HBr, to produce r-GO which has a maximum specific capacitance of 158 F g^−1^ at 0.2 A g^−1^ in BMI-PF_6_ (Chen et al., 2011). There are still some OCGs in such r-GO sample. The OCGs offer an affinity between r-GOs and water and hence enable the r-GO to disperse well in water. The lateral dimension of the thinnest r-GO sheet containing 2-3 graphene layers was found to be about 150–250 nm. Thus, the corrugation and scrolling may exist simultaneously on the r-GO sheets. This r-GO material was capable of a better capacitive performance in the aqueous electrolyte than BMI-PF_6_. The specific capacitance of r-GO at the same specific current reached to 348 and 158 F g^−1^ in aqueous and BMI-PF_6_ electrolytes, respectively. However, in a recent study of the thick r-GO supercapacitor electrodes, the supercapacitor using the BMI-BF_4_ electrolyte exhibited a better capacitive performance than the one using an aqueous electrolyte because the pores of the r-GO sample were precisely modified to accommodate the ions of the IL (Li et al., 2016). From the examples above, it can be noticed that the accessibility of IL electrolytes to the r-GO surface can greatly affect the capacitive performance of r-GO materials. Some more efforts have been made to increase the effective porosity and the accessibility of IL electrolytes by adding CNTs as spacers (Cheng et al., 2011; Lin et al., 2013; Pham et al., 2015) or using a special treatment (Yu et al., 2016b).

It is believed that the interposed CNTs in the inter-graphene spacing can prevent the graphene sheets to restack with one another. Researchers found that there was always an increase of specific capacitance of the CNT/r-GO composite (which is actually a hybrid material) after long time charging-discharging cycling (Cheng et al., 2011). They called this phenomenon electro-activation. The electro-activation occurs because the repeated charging-discharging cycling could help ions intercalate into the graphene layers where the electrolyte cannot fully access originally. After cycling, the inner-graphene spacing may be enlarged by the intercalation and the enlarged spacing can provide a larger effective surface area for capacitance. For the same CNT/r-GO composite, the specific capacitance can be increased by 18% after a long time cycling in an aqueous electrolyte, whilst the increasement was found to be 29% in the EMI-TFSI electrolyte (Cheng et al., 2011). Meanwhile, a supercapacitor using the CNT/r-GO composite electrodes and the EMI-TFSI electrolyte reached a specific energy of 155.6 Wh kg^−1^, which was very high when the result was published. In addition, the 3D structures of such CNT/r-GO composites do not only help increase the energy capacity, but also increase the power capability. In a recently published paper, it was claimed that a supercapacitor comprising two 3D CNT/r-GO (called CNT/graphene in original paper) composite electrodes and the EMI-BF_4_ electrolyte exhibited an outstanding capacitive performance. The maximum energy density of the cell reached 117.2 Wh L^−1^ (110.6 Wh kg^−1^ for specific energy) at the maximum power density of 424 kW L^−1^ (400 kW kg^−1^ for specific power) based on the active materials (Pham et al., 2015).

In addition to CNTs and r-GOs, the other porous carbon materials also play an important role in supercapacitors. High surface Act-Cs are the predominant electrode materials in the commercial supercapacitors because they are easy to produce. The Act-Cs produced by pyrolyzing polypyrrole has a specific capacitance up to 300 F g^−1^ in the EMI-BF_4_ electrolyte and, more interestingly, 5–8% of the performance improvement was achieved after 10,000 charging-discharging cycles at a high specific current of 10 A g^−1^ (Wei et al., 2012) (Note that in this and some other literatures, the unit of A g^−1^ was often incorrectly linked with current density, which should be corrected as specific current). When ILs were used as the electrolytes in supercapacitors, the pore and ion sizes and the accessibility of the IL ions to the surface of Act-Cs must be considered. The changes of microstructure were considered during the synthesis of the Act-Cs. It was concluded that the optimal pore size should be perfectly adapted to the size of ions for efficient adsorption, whilst larger or smaller pores would reduce the gravimetric capacitance (i.e., specific capacitance). Another work indicated that the sub-nanometre pores may slow down the ion diffusion rate and they didn't contribute the capacitance in the EDLCs using IL electrolytes (Largeot et al., 2008).

In fact, the precursors of Act-Cs are not limited to the artificial polymers, like polypyrrole mentioned above, various biomasses can be used to prepare the high-value Act-C materials. For example, natural silk was used to produce hierarchical porous nitrogen-doped carbon nanosheets through a one-step and facile large-scale synthesis route using ZnCl_2_ and FeCl_3_ as the effective activation-graphitization agents. The nanosheets exhibited a specific capacitance of 242 F g^−1^, an energy density of 48 Wh L^−1^ (102 Wh kg^−1^ for specific energy), and a capacity retention of 81% after 10,000 cycles in the EMI-BF_4_ electrolyte (Hou et al., 2015). The biomass algae, Enteromorpha, was used to synthesize nitrogen and oxygen co-doped hierarchical porous carbons (Yu et al., 2016a), which possessed a sponge-like 3D interconnected structure with combined macro-/meso-/micro-pores. The specific surface area of the carbons was estimated up to 2,073 m^2^ g^−1^. The supercapacitor comprising two sponge-like carbon electrodes and the EMI-BF_4_ electrolyte exhibited a specific capacitance of 201 F g^−1^ at 1 A g^−1^ at 20°C. Rice straws were also used to produce the porous carbons as supercapacitor electrode materials (Sudhan et al., 2017). Although the capacitive performance of these carbons in EMI-BF_4_ was not outstanding, this study proposed a feasible process dealing with the excess rice straws, which are the most common biowaste materials from different agriculture sectors. The porous carbons made from bamboo exhibited a remarkable specific capacitance of 192 F g^−1^ at 100 A g^−1^ whilst the corresponding EES device using an aqueous electrolyte only had a specific energy of 6.1 Wh kg^−1^ under the specific power of 26 kW kg^−1^ based on the active materials (Tian et al., 2015). However, when the aqueous electrolyte was replaced by an IL, EMI-TFSI, the energy capacity and power capability of the supercapacitor consisting of the same carbon electrodes were enhanced and the specific energy and power reached 43.3 Wh kg^−1^ and 42 kW kg^−1^, respectively.

Based on the examples given above, there are several keynotes for using IL electrolytes in supercapacitors: (1) a wider operating voltage range leading to a higher MCV, up to 4.5 V; (2) a possible increase of specific capacitance due to the controversial hypothesis of the accessibility of ILs to the porous carbons; (3) the carbon morphology, especially the macro/meso/microporous structure, affects the capacitive behavior significantly. Obviously, it must be noted that in addition to porous carbon materials, the other materials with similar macro/meso/microporous structure can also be used as the supercapacitor electrode materials. 2D microporous covalent triazine-based frameworks showed a great potential in IL-based supercapacitors, and the rational design of electrode structures *via* bottom-up strategies could help further understand the capacitive EES mechanisms as well as better design the capacitive EES devices (Hao et al., 2015). A highly porous diamond foam was introduced as a kind of promising electrode material for supercapacitors because of the unmatched potential windows of the boron-doped polycrystalline diamond in aqueous solutions (Gao et al., 2014) or ILs (Gao et al., 2015). The porous boron-doped polycrystalline diamond can grow on the silica or silicon substrates. Consequently, the silicon nanowires (SiNWs) with and without coating materials were used in on-chip supercapacitors where ILs were used as the electrolytes (Berton et al., 2014; Thissandier et al., 2014; Aradilla et al., 2015a,b, 2016a,b; Gao et al., 2015; Gaboriau et al., 2017). These micro-power source devices with IL electrolytes had the high MCVs up to 4.0 V, which can hardly be reached when the traditional organic electrolytes were used (Thissandier et al., 2012). Furthermore, these micro-supercapacitors can be integrated into the miniaturized devices which have been highly demanded by the development of micro-electronics. Some more details on micro-supercapacitors will be discussed in the latter section.

In fact, the studies of the electric double layer capacitance mechanisms have been done in the EDLC models consisting of the different carbon materials and ILs. Different from the traditional EDLC model, an electric double-cylinder capacitor (EDCC) model for microporous carbon materials in organic electrolytes was proposed to take the pore curvature into account when the model was simulated by using a DFT method (Huang et al., 2008). The simulation results based on the EDCC model are consistent with the results obtained from the molecular dynamic simulations of the porous CNT electrodes in ILs, such as EMI-BF_4_ (Shim and Kim, 2010) and EMI-TFSI (Ma et al., 2017). A further agreement between the experimental and simulation results was achieved when the realistic atomistic structure of micropores and the electrode atomic polarization caused by ionic charges were considered in a simulation work based on the model of CDCs in BMI-PF_6_ (Merlet et al., 2012). Meanwhile, the instinct characteristics of ILs as electrolytes were also studied to improve the understanding of the ILs which are considered as the charged interfaces (Gebbie et al., 2013, 2015). Differential capacitance is directly related to the capacity of EDLCs. A simulation of the electrode | IL interface was done to correlate the change of differential capacitance with surface potential (Vatamanu et al., 2010; Ma et al., 2014). It was also found that small ions, like Li^+^, in an IL solution will change the original ionic composition of the IL and construct different electrode | IL interfaces leading to a smaller differential capacitance compared with the one of the neat IL (Zhao et al., 2010).

### IL-Mixture Electrolytes for Supercapacitors

ILs are liquid salts and they can be used alone as the electrolytes in supercapacitors. Meanwhile, they can also be used as the supporting electrolytes in solvent-based electrolytes. As supporting electrolytes, ILs have the same function as the other salts, providing cations and anions in the organic solutions mostly for the charge transport. Theoretical and experimental studies have been focused on the relationship between the specific capacitance of porous carbons and their pore size, especially the capacitance contribution from micropores (Largeot et al., 2008; Jiang et al., 2011; Griffin et al., 2015; Zhu et al., 2016). IL Solutions comprising AN or propylene carbonate (PC) were the typical electrolytes used in previous research. The activated graphene was found to be useful for the EES devices and exhibited a specific capacitance of 200 F g^−1^ at 0.7 A g^−1^ in BMI-TFSI and 166 F g^−1^ at 5.7 A g^−1^ in the BMI-TFSI/AN electrolyte (Zhu et al., 2011). Although the specific capacitance in BMI-TFSI was larger than that in the BMI-TFSI/AN electrolyte, the CVs of the graphene in BMI-TFSI were distorted from the rectangular shape whilst the ones in the ILs/AN electrolytes were still rectangular. It was suggested that the large ion size of the ILs caused the high viscosity and large charge transfer resistance of the electrolytes, leading to the distorted CVs. Similar findings were also reported about the activated graphene in the EMI-TFSI/AN electrolyte (Kim et al., 2013; Zhang F. et al., 2013; Zhang L. et al., 2013) and the graphene sheets-cotton cloth composite in the EMI-BF_4_/AN electrolyte (Liu et al., 2012). A micro-supercapacitor made of graphene quantum dots exhibited a higher frequency response in the EMI-BF_4_/AN electrolyte in comparison with the ones in aqueous electrolytes (Liu et al., 2013).

It is perceived the IL-solvent mixtures keep a wider operational voltage range and a higher ionic conductivity compared to the neat ILs. Thus, they are the good candidates for the electrolytes of supercapacitors. However, the liquid temperature ranges of these mixtures are narrower than the ones of the corresponding ILs due to the existence the traditional solvent (Ruiz et al., 2012). It is necessary to identify the temperature limits before an IL-solvent mixture is utilized as the electrolyte of capacitive EES devises. In addition to the temperature range issue, the risk from the flammable organic solvent is also a challenge to the further applications of IL-solvent mixture.

The IL-mixture electrolytes are not limited to the mixtures of ILs and molecular solvents. A eutectic mixture of ILs can dramatically decrease the melting point or show no melting point but a glass transition. For example, N-propyl-N-methylpiperidinium bis(fluorosulfonyl)imide (PMPip-FSI) and N-butyl-N-methylpyrrolidinium bis(fluorosulfonyl)imide (BMPyrr-FSI) were mixed at the 1:1 wt. ratio to make a eutectic mixture, which was used as the electrolyte in supercapacitors (Tsai et al., 2013). The eutectic IL electrolyte keeps liquid in the investigated temperature range from −50 to 80 °C. When the temperature was increased from room temperature to 80°C, a supercapacitor comprising two activated graphene electrodes and the eutectic IL electrolyte exhibited a increasing specific capacitance up to 180 F g^−1^ but a decreasing MCV from 3.5 to 2.8 V.

**Figure 4:**
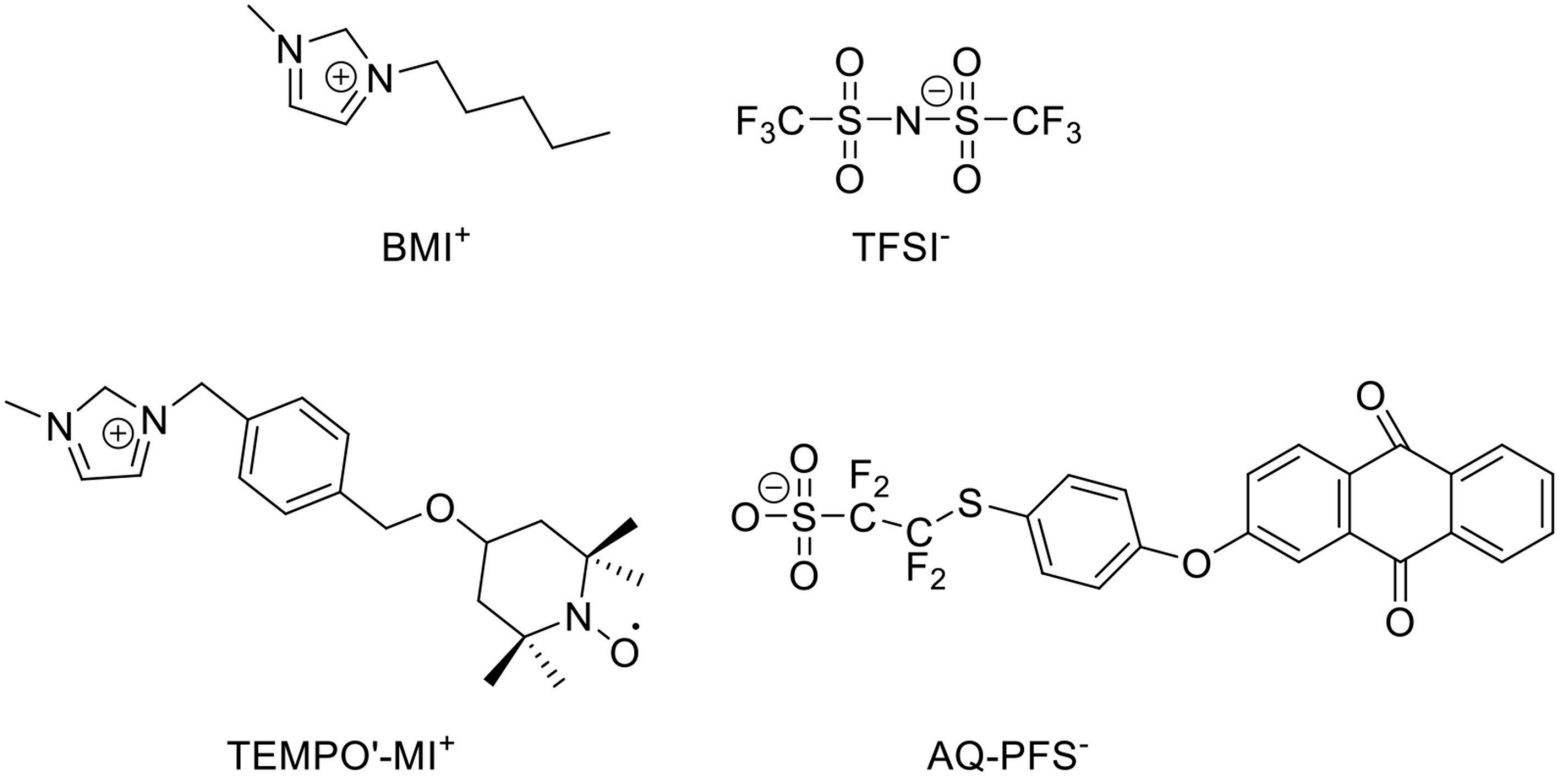
Structure of BMI-TFSI and the biredox IL with anthraquinone and 2,2,6,6-tetramethylpiperidinyl-1-oxyl group (Mourad et al., 2017).

In supercapacitors, ILs can also contribute to the energy capacity of capacitive EES devices. A recent report about the biredox ILs presented a good example. BMI-TFSI was mixed with a biredox IL, which comprises a perfluorosulfonate anion bearing anthraquinone (AQ-PFS^−^) and a methyl imidazolium cation bearing 2,2,6,6-tetramethylpiperidinyl-1-oxyl (TEMPO^•^-MI^+^) (Mourad et al., 2017). This IL mixture was used as a supercapacitor electrolyte. Figure 4 shows the molecular structures of the ILs. When the supercapacitor was charging, the cations and anions of BMI-TFSI were drawn into the negatrode and positrode, respectively, and they were adsorbed on the carbon surface without invoking any Faradaic reaction. In contrast, when the redox-active AQ-PFS^−^ and TEMPO^•^-MI^+^ were electro-adsorbed on the surface of the carbon electrodes, they underwent fast faradaic reactions. It is perceived that a dissolved redox species in electrolytes can result in a serious self-discharging problem in EES devices, especially supercapacitors. The mixture of the biredox IL and BMI-TFSI solved this problem because of their bulky size and high viscosity.

**Figure 5:**
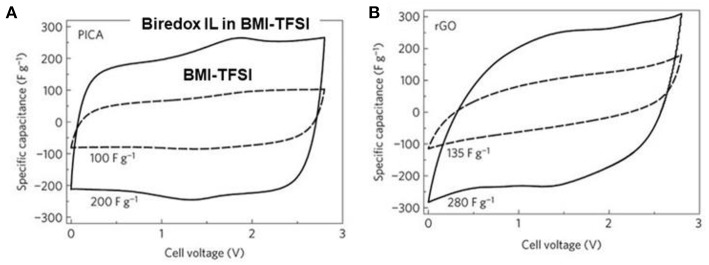
CVs of supercapacitors made of PICA (Act-Cs) **(A)** and r-GO **(B)** at 5 mV s^−1^ with 0.5 mol L^−1^ biredox IL in BMI-TFSI (solid line) and pure BMI-TFSI (dash line), respectively (Mourad et al., 2017). (Reprinted with permission from Springer Nature. Copyright 2016).

To study the biredox IL mixture in supercapacitor, Act-Cs (PICA) and r-GO were chosen as the electrode materials. Figure 5 presents the CVs of the carbon-based supercapacitors with 0.5 mol L^−1^ biredox IL in BMI-TFSI and pure BMI-TFSI at 5 mV s^−1^. PICA contained both micro- and mesopores, while r-GO offered an open surface with theoretically unrestricted access for the IL ions. In the CVs of the two carbons, the current amplitude doubled when the electrolyte was changed from BMI-TFSI to 0.5 mol L^−1^ biredox IL electrolyte. Broad oxidation and reduction peaks were observed at the intermediate voltages, indicating the redox processes of the biredox IL. In summary, the biredox IL was found to be able to increase the energy capacity of supercapacitors, by storing a significant amount of charge and retaining the redox species in the pores of electrodes. More detailed discussion about the contribution to energy capacity from the redox electrolytes can be found in the literature (Akinwolemiwa et al., 2015).

## Supercapatteries

The discussions above are mainly related to the IL-based electrolytes for supercapacitors, particularly EDLCs. Theoretically, there is no redox reaction involved in the charge storage of EDLCs. Thus, only the specific capacitance of electrode materials and the MCVs should be considered when calculating the specific energy of EDLCs. The IL-based electrolytes are favored in EDLCs because the MCVs is usually equal to the decomposition voltages of electrolytes and hence the wide operating potential windows of ILs are beneficial to the specific energy of EDLCs as we discussed in section Supercapacitors. The modified or doped materials were also used for supercapacitors and they had a higher specific capacitance than the un-modified or un-doped ones because of the redox activity induced by the modification or doping in the materials. Transition metal oxides (TMOs) and electronically conducting polymers (ECPs) are another typical pseudocapacitance materials because of their redox nature. Although the specific capacitance of these pseudocapacitance materials are higher than the one of EDLCs, the potential ranges of these pseudocapacitance materials are usually narrower than 1.0 V. In this case, the symmetrical devices made of pseudocapacitance materials are not favorable for high energy capacity EES devices and the asymmetrical devices have been proposed to achieve high voltage. There are two main designs of asymmetrical devices. One is so called asymmetrical supercapacitors, which were first proposed and constructed by the positrode and negatrode capable of capacitive charge storage, typically the permutation and combination of the EDL and pseudocapacitance electrodes. The other design of asymmetrical devices is using a hybrid configuration that combines a supercapacitor electrode and a battery electrode into one device. Such hybrids have been reported under different names which are mainly corresponding to the different electrode materials. The word hybrid is obviously not a suitable unified expression for the future development of these asymmetrical devices as it is too abstract whilst supercapattery or supercabattery can be a general term to represent these asymmetrical devices vividly. In general, supercapattery takes advantages of the Faradaic charge storages typically the non-capacitive Faradaic store. Thus, pseudocapacitors fall in the scope of supercapatteries in a broad sense. More often, the battery-type storage should be involved in supercapatteries.

**Figure 6:**
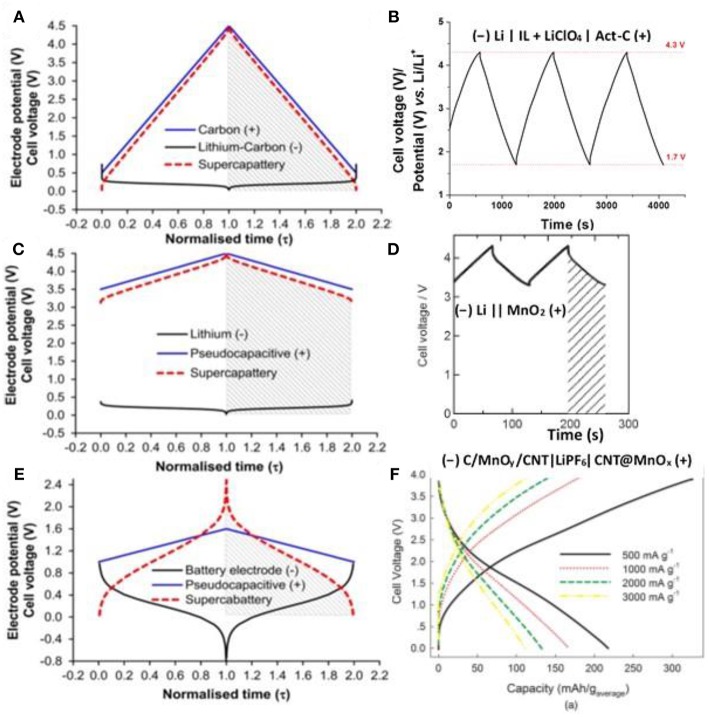
Calculated electrode potential (black and blue lines for negatrode and positrode) and cell voltage (red dashed lines) as a function of normalized time for galvanostatic charging and discharging (GCD) of three types of hypothetical supercapattery and the GCD plots of the related experimental demonstration of supercapatteries. **(A)** a hypothetical supercapattery with a negatrode of lithium metal or lithiated carbon and a positive positrode of activated carbon; **(B)** an experimental demonstration of **(A)** (–) Li | IL + LiClO_4_ | Act-C (+) (Yu and Chen, 2016a); **(C)** a hypothetical supercapattery with a negatrode of lithium metal or lithiated carbon and a pseudocapacitive positrode; **(D)** an experimental demonstration of **(C)** (–) Li | PEO-LiTFSI | LTAP | 1.0 M LiCl aq. | MnO_2_ (+) (Makino et al., 2012) (Reprinted with permission from the Royal Society of Chemistry. Copy right 2012); **(E)** a hypothetical supercapattery with a negatrode of the typical battery type and a pseudocapacitive positrode; and **(F)** an experimental demonstration of **(E)** (–) C/MnO_y_/CNT | LiPF6 | CNT@MnO_x_ (+) (Zhou et al., 2016) (Reprinted with permission from the Electrochemical Society. Copyright 2016). **(A)**, **(C)** and **(E)** are adapted from the reference (Chen, 2017).

First, we hypothesized a supercapattery with a negatrode of lithium (Li) metal and a positrode of Act-Cs and then predicted the behavior of the electrodes and the cell as shown by the galvanostatic charging and discharging (GCD) plots in Figure 6A. The behavior of the cell is highly capacitive and hence Equation (1) is still valid to calculate the energy capacity of the hypothetic cell. The minimum potential of the Act-C electrode was set to 0.5 V *vs*. Li/Li^+^ to prevent the lithiation of the Act-C electrode during discharge.

The balance of the electrode masses or charges is important for all the EES devices. The charges passed through the positrode and negatrode in a supercapattery must be the same. In this case the masses of the positrode and negatrode were fixed according to the charges passed through as demonstrated in Equation (2).

(2) $$\begin{array}{r} Q_{-}=m_{-}Q_{sp-}=m_{+}C_{sp+}\Delta E_{+}=Q_{+} \end{array}$$

where *Q* represents the charge, *m* the mass of the electrode, *C*_sp_ the specific capacitance of the electrode, Δ*E* the potential range, and the subscript _+_ and _−_ the positrode and negatrode, respectively. In the case of the lithium metal negatrode described in Figure 6A, *Q*_sp, Li_ = *nF*/*M*_Li_ = 13900 C g^−1^ = 3861 mAh g^−1^, where *n* = 1, *F* = 96485 C mol^−1^, and *M*_Li_ = 6.941 g mol^−1^. As to the Act-C positrode, we hypothesis *C*_sp, C_ = 200 F g^−1^ and Δ*E* = 4.0 V. After rearranging Equation (2) and filling in all the data above, we calculated the mass ratio of the positrode and negatrode, $m_{C}/m_{Li}=Q_{sp,Li}/C_{sp,C}\Delta E=\frac{13900}{200\times 4.0}=17.4$. When the total mass of this supercapattery was evaluated, the mass of the lithium metal negatrode was negligible compared to that of the Act-C positrode. In this case, the capacitance of the cell is equal to the one of the Act-C positrode approximately. Because the minimum potential of the Act-C electrode was set to 0.5 V *vs*. Li/Li^+^, instead of zero, Equation (1) must be modified to Equation (3).

(3) $$W_{\max}=\frac{1}{2}C(U_{\max}^{2}-U_{\min}^{2})$$

Taking *C* ≈ *C*_*sp, C*_ = 200 F g^−1^, *U*_max_ = 4.5 V and *U*_min_ = 0.5 V into Equation (3), we calculated the specific energy of the hypothetic cell that *W*_max_ = 555.6 Wh kg^−1^.

On the experimental side, a supercapattery consisting of an Act-C positrode, a Li/Li^+^ negatrode, and an IL electrolyte of 1-butyl-1-methylpyrrolidinium tri(pentafluoroethyl)trifluorophosphate (BMPyrrFAP) with dissolved gamma-butyrolactone (γ-GBL) and LiClO_4_ was successfully demonstrated. The IL solution did not only provide cations and anions for non-Faradaic capacitive storage at the Act-C surface, but also enable the Li/Li^+^ redox reaction on the negatrode for non-capacitive Faradaic or Nernstian storage. The GCD plot of this supercapattery is shown in Figure 6B, demonstrating a typical capacitive charging and discharging feature. The specific energy of the supercapattery reached 230 Wh kg^−1^ at a GCD current density of 1 mA cm^−2^ (based on active materials), which was the highest record for supercapatteries using Act-Cs as the electrode materials (Yu and Chen, 2016a).

When constructing the second hypothetic supercapattery as shown by the GCD plots in Figure 6C, we replaced the EDLC positrode by a pseudocapacitive electrode whose specific capacitance is higher than EDLC e.g, *C*_sp_ = 500 F g^−1^, whilst the potential range is narrower e.g. Δ*E* = 1.0 V. The mass ratio of the positrode and negatrode is $m_{C}/m_{Li}=Q_{sp,Li}/C_{sp,C}\Delta E=\frac{13900}{500\times 1.0}=27.8$, which is also big enough to neglect the mass of the lithium metal negatrode when evaluating the energy capacity as we calculated above. Similarly, taking *C* ≈ *C*_*sp, C*_ = 500 F g^−1^, *U*_max_ = 4.5 V and *U*_min_ = 3.5 V into Equation (3), we calculated the specific energy capacity of the second hypothetic cell that *W*_max_ = 555.6 Wh kg^−1^.

Some recent studies on the cell of (−) Li | PEO-LiTFSI | LTAP | 1.0 mol L^−1^ LiCl (60 °C) | MnO_2_ (+) (Makino et al., 2012), where PEO-LiTFSI is a buffer layer and LTAP is a solid electrolyte made of LISICON-type solid glass ceramic, presented the GCD plot as shown in Figure 6D which is very comparable with the calculated plot shown in Figure 6C.

The lithium metal electrode works reversibly at the most negative potential among all the battery electrode materials. In contrast, the sluggish GCD plots can be observed for most battery electrodes as demonstrated in Figure 6E. Similar to the second hypothetic supercapattery, a pseudocapacitance positrode and a battery negatrode was combined to fabricate the third hypothetic device. The battery negatrode in the third hypothetic device works at more positive potential and its GCD is more sluggish than the lithium metal negatrode. It can be observed from Figure 6E that the GCD plot of the third hypothetic device is not a straight line in either charging or discharging, and hence it does not represent a simple capacitive behavior. As a result, we had to integrate the GCD of the cell to evaluate the cell energy capacity, instead of using Equation (3). The shadows shown in Figure 6E cover the area under the discharging branch of the GCD plot and the shadow area is proportional to the energy capacity of the cell. We can notice that the behavior of the cell is more like a battery and supercabattery is a more proper term for the cell.

The GCD plot of a typical example of supercabattery is shown in Figure 6F. ACNT@MnO_x_ was synthesized by the reaction of ACNTs and KMnO_4_ and the composite was used as the positrode material demonstrating a capacitive behavior in the supercabattery. ACNT@MnO_x_ was further coated by the carbons using CVD in C_2_H_2_ atmosphere to produce C/MnO_y_/ACNT, which was used as the negatrode materials in the supercabattery. The supercabattery reached a specific energy up to 208.6 Wh kg^−1^ and remained 105.8 Wh kg^−1^ under an ultrahigh specific power of 3,000 W kg^−1^ (Zhou et al., 2016).

According to the discussion above, the concept of supercapattery can easily clarify the Faradaic storage in the specified capacitive EES devices. For now, the expression of supercapacitor or hybrid device can be easily found from the literatures for the capacitive EES devices possessing Faradaic charge storage (Lukatskaya et al., 2016; Chen, 2017). Supercapattery is gradually accept by the EES community (Chen, 2017), and the number of the papers on supercapattery increased exponentially in the past 5 years. However, the aqueous or organic electrolytes were still used for the most reported supercapattery demonstrations and the typical examples of supercapattery using IL electrolytes are still rare. Consequently, only one supercapattery demonstration using the lithium metal negatrode, Act-C positrode and IL-based electrolyte was exemplified in Figure 6B. Another two experimental devices as exemplified in Figures 6D,F were based on the aqueous/solid hybrid electrolyte and organic electrolyte, respectively.

In the example of Figure 6B, the IL-mixture electrolyte does not only supply the Li^+^ for the redox reaction related to the Faradaic charge storage, but also possess lower viscosity than the neat IL. This strategy was also applied in another reported supercapattery using EMI-TFSI/AN as the IL-mixture electrolyte (Ortaboy et al., 2017). In this study, manganese oxide-decorated carbonized porous silicon nanowire (MnO_x_/C/PSiNWs) arrays were used as the Faradaic storage positrode materials, while PSiNWs as the EDL negatrode materials. The supercapattery (described as hybrid in the published paper) exhibited an excellent specific power of 25 kW kg^−1^ and specific energy of 261 Wh kg^−1^ at a current density of 0.2 mA cm^−2^ within 3.6 V. The capacity retention of the device kept higher than 80% after 10,000 CV cycles. It is a promising result, whilst the challenge of these electrolytes remains that there is still a volatile organic solvent in the electrolyte.

It should be mentioned that the Faradaic charge storage can also be obtained by dissolving a redox component in the electrolytes of a supercapacitor, typically EDLC as mentioned in section IL-Mixture Electrolytes for Supercapacitors. A cell using the biredox IL electrolyte demonstrated an increase of the energy capacity by adding the redox IL in another IL as shown in Figure 5. Because both Faradaic charge storage and EDL storage are exist in one device, this cell is also a special type of supercapattery.

## Micro-Supercapacitors

Micro-supercapacitors fall in the scope of supercapacitors and supercapatteries in terms of the charge storage mechanism and the choices of electrode materials and electrolytes. The energy capacity and power capability of these miniaturized EES devices do not only depend on the choices of materials but also the topology or configuration of the devices. In general, a supercapacitor or supercapattery has a sandwich structure, whilst a micro-supercapacitor is a miniaturized EES device standing on a substrate of several square millimeters. As discussed in the previous section, the energy capacity and power capability of supercapacitors or supercapatteries are generally evaluated by the relevant gravimetric values. This strategy is not very useful for micro-supercapacitor because these small size devices are surface dependent. The areal metrics are more popular for micro-supercapacitors. Quite a lot of supercapacitor electrode materials have been utilized in micro-supercapacitors. Although it is a challenge to fabricate the electrode materials on a substrate of tiny area, the main technological barrier restraining the transfer of micro-supercapacitors from laboratory demonstration to pilot production is still the electrolytes issue (Lethien et al., 2019).

**Figure 7:**
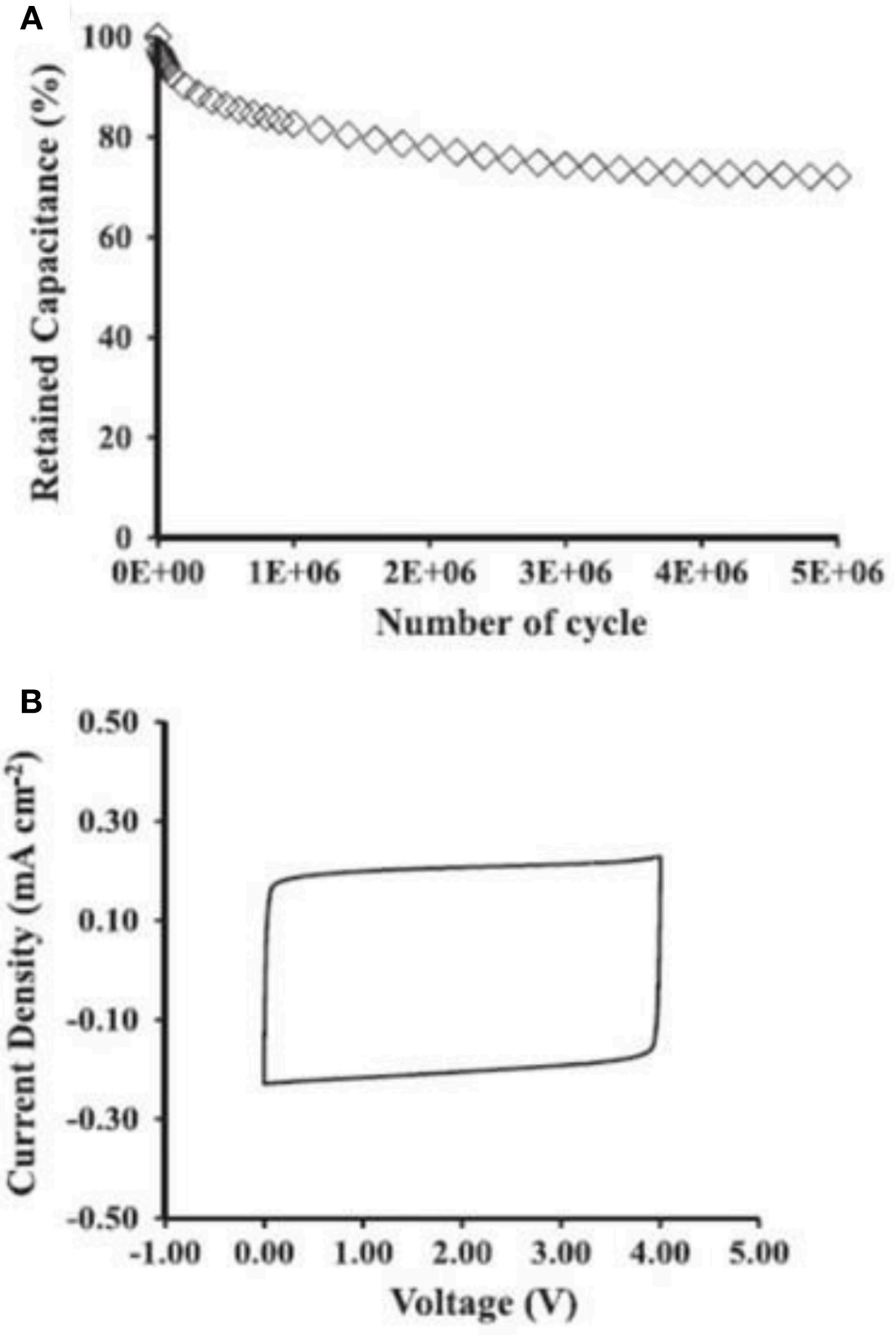
Performance of the SiNWs micro-supercapacitors: **(A)** plots of capacitance retention ratio against the complete GCD cycles at 2 mA cm^−2^ between 0 and 4 V. **(B)** CVs after the GCD test at 20 V s^−1^ between 0 and 4 V (Aradilla et al., 2015b). (Reprinted with permission from Aradilla et al. (2015b) under the terms of the Creative Commons Attribution 3.0 license).

The energy capacity is always the first consideration of supercapacitors, so do micro-supercapacitors. When ILs are used as the electrolytes in micro-supercapacitor, the MCV can be increased up to the decomposition voltages of ILs. Figure 7 shows the performance of a micro-supercapacitor made of the SiNWs electrodes and an IL, triethylammonium bis(trifluoromethylsulfonyl)imide. The SiNWs are ~35 μm in length. It was found that the micro-supercapacitor only lost 27% of the original capacitance after 5 × 10^6^ complete GCD cycles in a voltage range from 0 to 4.0 V as shown in Figure 7A. After the GCD, the micro-supercapacitor was further examined by cyclic voltammetry. The CVs kept rectangular as shown in Figure 7B demonstrating an outstanding stability of the micro-supercapacitor.

Micro-supercapacitors with IL electrolytes possess high energy capacity due to the wide operating voltage of ILs. In practice, these on-chip supercapacitors would be integrated in the miniaturized devices, especially the wearable and portable devices. Thus, all-solid-state micro power sources devices would be favorable.

All-solid-state EES devices are gradually attracting attentions from both academia and industry, because they do not require high standard safety encapsulation materials compared with the other EES devices using liquid electrolytes. Consequently, their geometric shape can be variable, which is favored by designers and customers. High conductive solid electrolytes are the most essential component in all-solid-state EES devices. At present, solid electrolytes based on pure oxides and polymers are still suffered by their low or ultralow ionic conductivity at room temperature.

**Figure 8:**
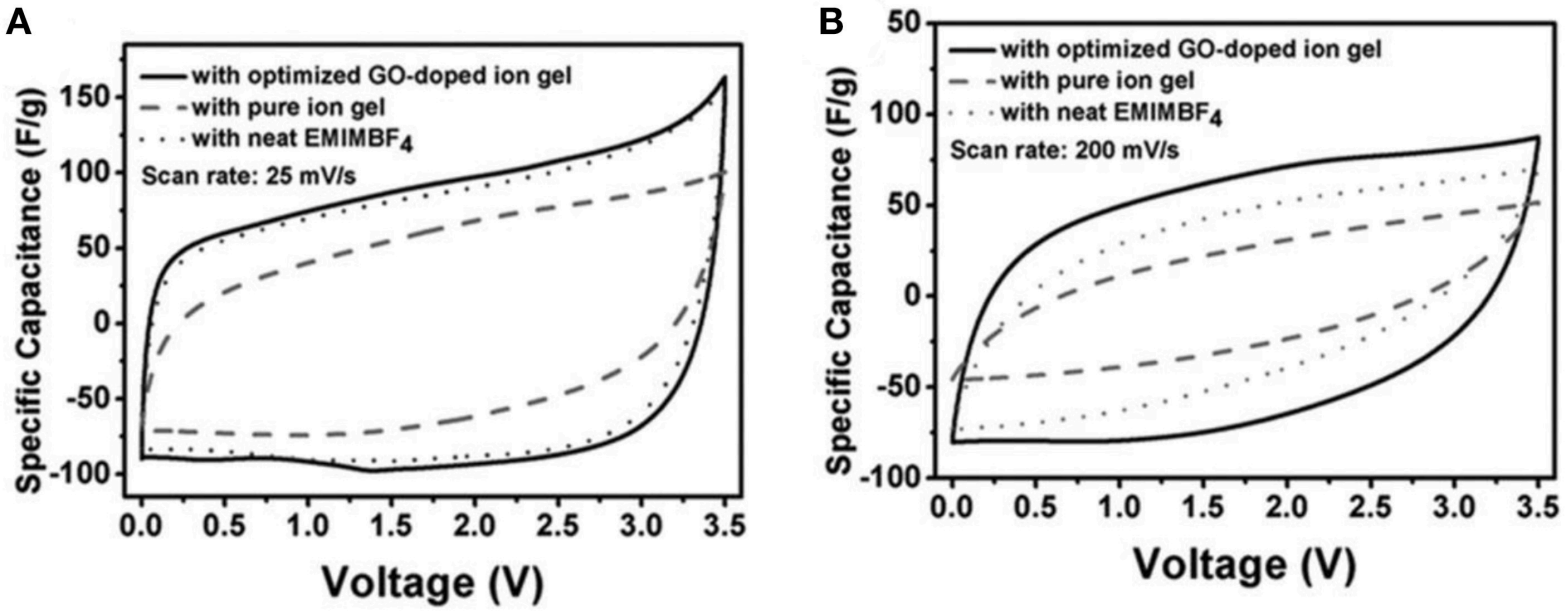
Comparison of CVs from all-solid-state supercapacitors with GO-doped ion gel and pure ion gel, and conventional supercapacitor with neat EMI-BF_4_ at a scan rate of 25 mV s^−1^ **(A)** and 200 mV s^−1^ **(B)**, respectively (Yang et al., 2013). (Reprinted with permission from John Wiley and Sons. Copyright 2013).

Many efforts have been dedicated to gel polymer electrolytes (GPEs), where ions are conducted through a polymer matrix. In a reported all-solid-state flexible supercapacitor, bacterial nanocellulose, CNTs, and an IL, EMI-TFSI, were used to fabricate an IL-based GPE. The copolymer consisting of bacterial nanocellulose and CNTs was used to make the electrodes. The specific capacitance was estimated up to 50 F g^−1^ based on the CVs of the all-solid-state flexible supercapacitor (Kang et al., 2012). In another work, GO was regarded as an ionic conduction promoter for the GPE made of EMI-BF_4_ and poly(vinylidene fluoride-hexafluoro propylene) [P(VDF-HFP)]. The demonstrated GO-doped GPE exhibited higher ionic conductivity than the pure GPE (Yang et al., 2013). The promoted ionic conductivity was attributed to the decrease of crystallinity in the GO-doped GPE compared with the pure GPE. This explanation was further proved by the differential scanning calorimetry data. It was also suggested that the degree of crystallinity in the GO-doped GPE should not be further reduced with more mass fraction of GO because a higher GO content was expected to deteriorate the ionic conductivity due to the restacking of GO sheets and the blocking effect by the excessive GO. Figure 8 compares the CVs from these all-solid-state supercapacitors with the GPEs (indicated as ion gel in the figure) with and without GO, and the conventional supercapacitor using neat EMI-BF_4_ at two scan rates. It was found that the CV from supercapacitor with GO-doped GPE was less distorted when the scan rate was increased, indicating the GO-doped GPE had lower internal resistance than the other two.

**Figure 9:**
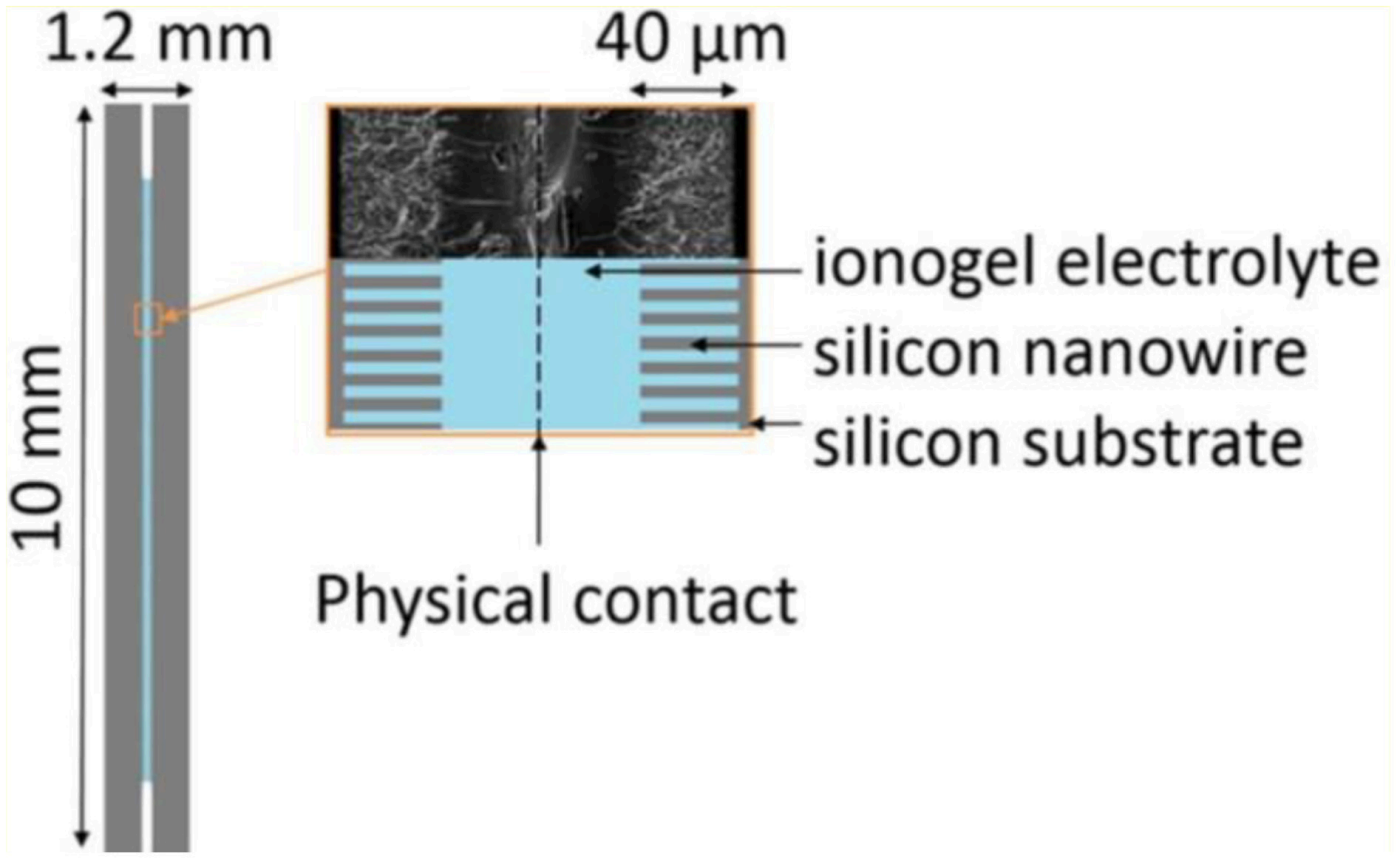
Schematic of the solid-state micro-supercapacitor and SEM image of the SiNWs electrodes covered by ionogel (Brachet et al., 2016). (Reprinted with permission from The Royal Society of Chemistry. Copyright 2016).

Ionogels resulted from a confinement of ILs in silica-like networks were also utilized as the solid electrolytes in micro-supercapacitors. The scan rate of the CVs of micro-supercapacitors can reach 10 V s^−1^, which demands a high ionic conductive electrolyte. An ionogel comprising an IL, EMI-TFSI, resisted a solder reflow which is an important process in the fabrication of micro-electronic devices (Brachet et al., 2016). Figure 9 shows the schematics of a micro-supercapacitor and the SEM image of a SiNWs electrode covered by a layer of the ionogel electrolyte. The ionogel layer was fabricated on the top of the SiNWs as seen from the SEM image in Figure 9. In this case, there is no need to add a membrane separator or fill a liquid electrolyte in the micro-supercapacitor. It was found that the all-solid-state micro-supercapacitor consisted of the silicon nanowire electrodes and the ionogel electrolyte exhibited the same capacitive performance as the one using the IL electrolyte. The ionogel have been proofed to be applicable in various micro-supercapacitors made of different electrode materials, like CNTs (Hsia et al., 2014), photoresist-derived porous carbons (Wang et al., 2014), etc.

Ionogels are not limited to the silica networks as described above. The other nano-porous solids with large surface area and strong surface interaction to ILs can also be used as matrix materials, such as titanium oxide, alumina, CNTs, GO, etc. In all the EES devices, the non-conductive matrix materials are favorable in ionogels because the electronic conductive materials may cause some serious problems, like short-circuit and self-discharge . The ionic conductivity and stability of ionogels and the wettability between ionogels and electrode materials should also be considered in the choice of matrix materials and ILs.

## Prospects

**Figure 10:**
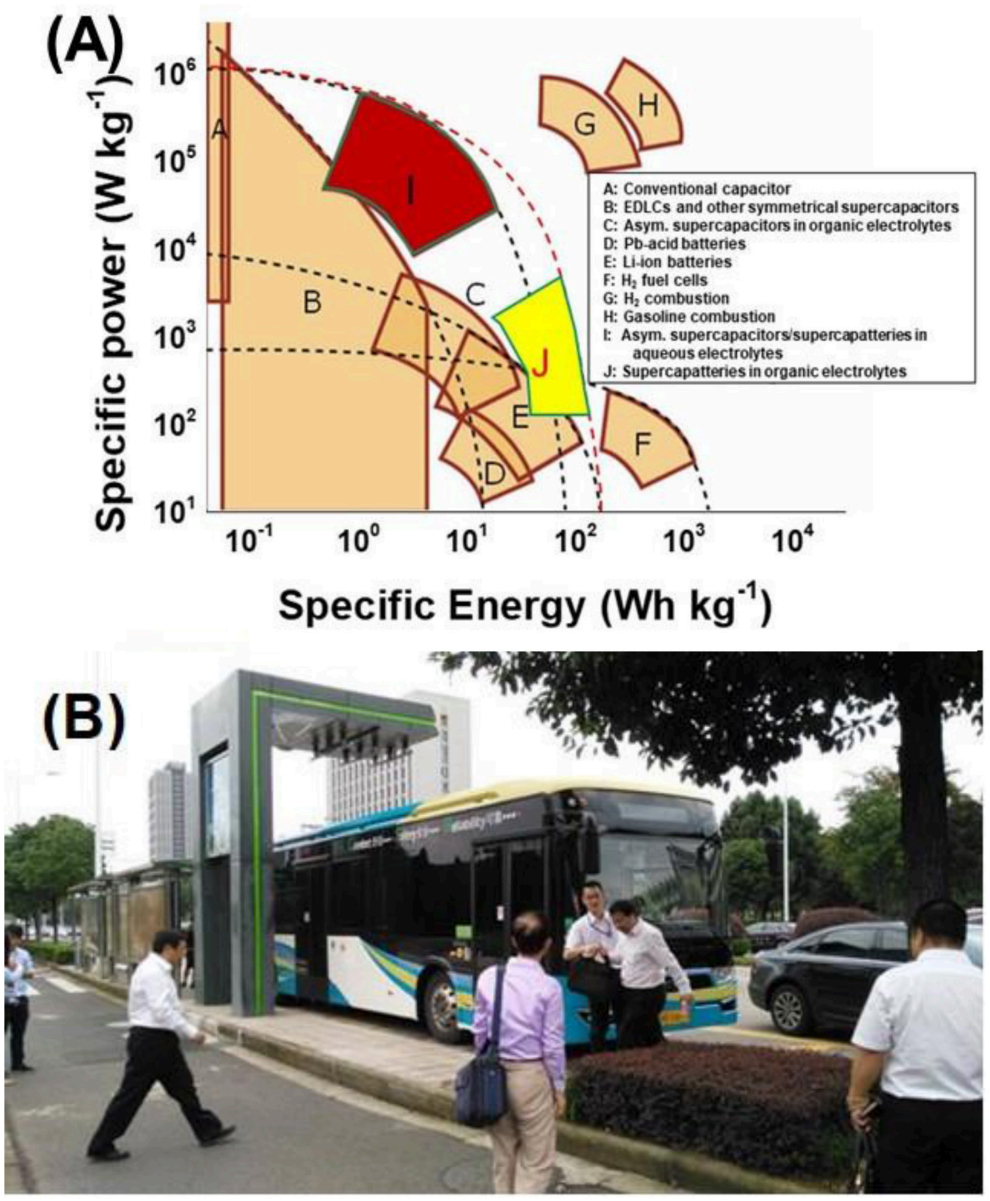
Ragone plots of various EES and internal combustion power devices **(A)** (Yu and Chen, 2016b), and a photograph of the supercapacitor powered electric bus at the road side stop which also serves as the charging station in Ningbo, China **(B)** (Akinwolemiwa et al., 2016).

In this review, we have introduced the recent progress on the capacitive EES devices from the perspective of IL electrolytes. The capacitive EES devices include supercapacitor, supercapattery and micro-supercapacitor (or EDLC, supercapattery and micro-supercapacitor from the view of charge storage mechanism) as described here and in the other reviews (Akinwolemiwa et al., 2015; Yu and Chen, 2016b; Chen, 2017; Xia et al., 2017). ILs have catered the trend for developing the high energy capacity EES devices. As described in the previous review on the redox electrode materials for supercapattery (Yu and Chen, 2016b) and shown in the Ragone plots of various electrochemical and internal combustion power devices from (Figure 10A), EDLCs have remained advantageous in terms of power capability, although they are still fallen behind the other EES devices when the values of specific energy are considered. Supercapatteries including pseudocapacitors and capacitive EES hybrids using aqueous electrolytes have also offered the high-power capability, while the organic electrolyte supercapatteries (block J in Figure 10A) are approaching the Li-ion batteries. The IL electrolytes have played an important role as a kind of organic electrolytes in this progress as introduced in this review. ILs containing large cations and anions may have some drawbacks when charge transport must be considered in some cases where high-power outputs are needed, while these drawbacks can be reasonably utilized to promote the energy capacity of capacitive EES devices, such as those supercapacitors using the biredox ILs. Supercapatteries using IL-based electrolytes are gradually becoming competitive compared with other EES devices when high-performance energy storage devices are strongly required nowadays. Following the continuous development in electrode materials for the capacitive EES devices, more and in-depth research efforts in the development of advanced electrolytes, especially those based on ILs, are needed to better understand the mechanism and kinetics of charge storage processes at the electrode | IL electrolyte interface for the technological advancement. The established market and public awareness can also stimulate the research on next generation of capacitive EES devices using IL electrolytes. On one hand, electric buses using supercapacitors (organic electrolytes) as the main power source have been serving the inner-city public transportation in Ningbo as shown in Figure 10B since 2015. On the other hand, the price for making and using ILs have been gradually reduced to an acceptable level along with the increasing demand from the global market. All progresses and efforts made from the research and industrial communities so far are promising a bright future for supercapacitor and supercapattery, and the fundamental research on IL-based electrolytes and the relevant technological innovation would be essential for the future development of supercapacitor and supercapattery.

## Author Contributions

All authors listed have made a substantial, direct and intellectual contribution to the work, and approved it for publication. GC supervised the work.

### Conflict of Interest Statement

The authors declare that the research was conducted in the absence of any commercial or financial relationships that could be construed as a potential conflict of interest.
